# Nutrition-Related Messages Shared Among the Online Transgender Community: A Netnography of YouTube Vloggers

**DOI:** 10.1089/trgh.2019.0048

**Published:** 2019-12-13

**Authors:** Heather E. Schier, Whitney R. Linsenmeyer

**Affiliations:** Department of Nutrition and Dietetics, Saint Louis University, St. Louis, Missouri.

**Keywords:** netnography, nutrition, qualitative research, social media, transgender, video blogs

## Abstract

**Purpose:** Nutrition care guidelines for the transgender population do not exist, despite significant nutrition-related clinical and psychosocial considerations. Social networking sites (SNSs) provide multidirectional communication and have expanded in popularity among transgender users as a resource for health information and support. The nature of the content shared among the online transgender community is unknown, but may suggest the nutrition-related areas that are of most importance to the transgender population. The objective of this qualitative netnography was to describe the food and nutrition messages shared among the transgender community using video blogs (vlogs) on the SNS, YouTube.

**Methods:** Public vlogs were assessed using the constant comparative method. Pseudoquantitative methods were used to capture the prevalence of each subtheme; quotes were documented verbatim. Data were collected from transgender users' public vlogs (*n*=30) self-published on YouTube from 2013 to 2018.

**Results:** Six major themes were generated from the data analysis. These included the following: functions of diet and exercise; diet and exercise philosophies; “how to” vlogs; advice for success; using dietary supplements; and effects of hormone therapy.

**Conclusions:** Nutrition-related messages are widely shared among the online transgender community through YouTube. The identified themes reflect topics of interest and expressed needs of transgender individuals. SNSs provide health care providers with a platform to improve patient education and health literacy. Health care providers may actively engage in online discussions to build trust, answer questions, and provide a source of accurate and evidence-based information.

## Introduction

An estimated 0.6% of adults, or 1.4 million people, identify as transgender in the United States. This figure has approximately doubled in the past decade.^1^ Nutrition care guidelines for the transgender population does not exist, despite significant nutrition-related clinical and psychosocial considerations. Transgender patients may experience changes in weight status and body composition, altered lipid levels, hypertension, and changes in bone mineral density second to hormone therapy and gender-affirming surgeries.^2–5^ Transgender patients have also reported elevated rates of body dissatisfaction, disordered eating, compensatory behaviors, and self-reported eating disorders compared with the cisgender population.^6–13^

### Social media as a health information resource

The transgender population faces several barriers to adequate health care, including discrimination, lack of trained providers, financial constraints, and unwelcoming physical facilities.^14^ As a result, the Internet has become a popular place for the transgender community to seek health-related information. Social media in particular has emerged as a platform for peer-to-peer education, resource sharing, and formation of a virtual social network.^15–19^ Transgender adolescents are utilizing social media to connect with a virtual transgender community, share personal experiences of transitioning, and learn about both medical and nonmedical transition strategies. However, these sites have also become platforms for bullying and discrimination.^18^

Given the pervasiveness of social media, scholars from the disciplines of social work, adolescent medicine, and public health have suggested active participation in virtual conversations by both patients and health care providers. Social media engagement provides an opportunity to understand the lived experiences of transgender individuals, build trust between the transgender community and medical providers, and engage trans voices while allowing for a level of anonymity when desired.^15^ Providers may engage in online forums in a question and answer format, and may also utilize social media campaigns to share positive health messages.^15,18^ Finally, active engagement in social media may be utilized to track the spread of inaccurate or misleading health information and provide an opportunity to debunk health myths.^15^

### Social networking sites as a data source

Social networking sites (SNSs) as a data source affords both advantages and disadvantages. Platforms offer researchers an accessible and affordable source for large data sets, can be used to describe hard to reach populations, and provide an opportunity for multidirectional communication where participants may express vulnerabilities more freely. Limitations are the primarily observational nature of most published studies, fluctuations in usage and popularity of various sites, and a skew toward younger and more technology-savvy participants.^20^ For the transgender population, online samples are typically younger and may not be reflective of older generations and gender identities.^15,20,21^

In particular, qualitative methods utilizing social media as a data source are gaining recognition because of unprecedented access to topical conservations and personal accounts.^15,22^ Although quantitative research has made good use of social media as big data, qualitative research utilizing SNSs remains an underused opportunity.^22^ Usage of publicly available blogs and SNSs of transgender individuals may even provide an opportunity to amplify the voices and perspectives of the transgender community.^23^

### Study purpose and aims

The purpose of this study was to explore the nutrition-related messages shared among the virtual transgender community. The study aims are to (1) describe the nutrition-related messages shared by transgender individuals through the video platform YouTube and (2) suggest the nutrition-related topics that are of greatest importance to the transgender community.

## Materials and Methods

### Study design

This ethnography is organized and reported according to the American Psychological Association Journal Article Reporting Standards.^24^ Ethnographic methodology within nutrition and dietetics research provides valuable insight on complex health issues and health inequalities.^25^ A netnography is a virtual or online ethnography that utilizes computer-mediated communications as the data source.^25,26^ Online video blogs (vlogs) were chosen as the data source given their expression of one's lived experience and the potential to amplify the voices of the transgender community.^27^ The underlying hermeneutical perspective was extended from its traditional application with written narratives to a modern application with verbal narratives that were recorded and self-published by individuals on the public domain, YouTube.

### Sampling and data collection

**FIG. 1.**
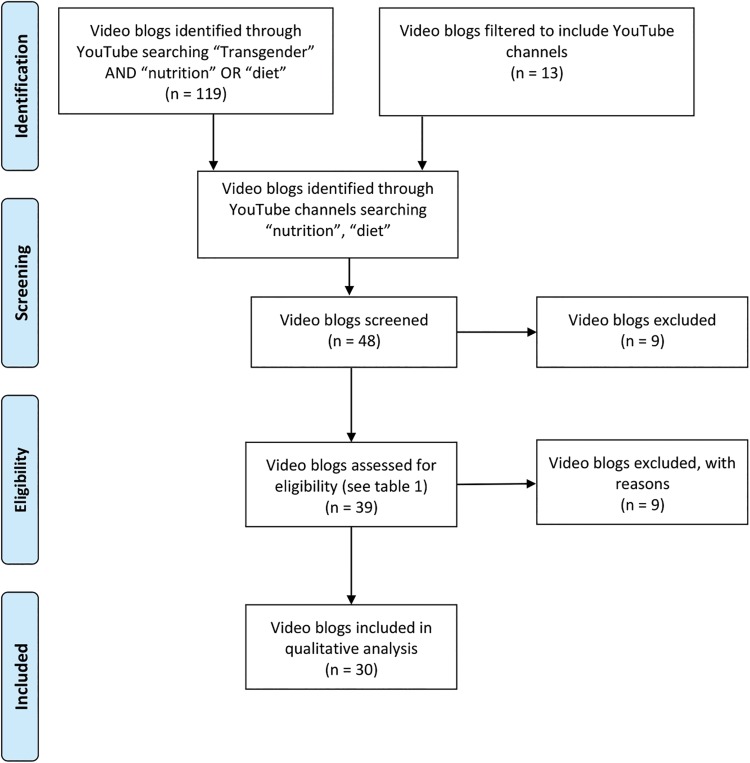
Sample selection of nutrition-related video blogs by transgender individuals through YouTube. Nutrition-related messages were transcribed and analyzed using the constant comparative method by two authors, separately.

A YouTube search (July 5, 2018) was used to identify relevant vlogs using the search terms “transgender,” AND “diet” OR “nutrition” (Fig. 1). YouTube filters were applied, including “channel.” This search resulted in 119 channels. Channels with fewer than 500 subscribers or individual videos with <1000 views were excluded. Individual channels were searched using terms “diet” and “nutrition.” The resulting videos were reviewed using inclusion and exclusion criteria detailed in Table 1, which resulted in 39 remaining videos sourced from 13 channels. The remaining 39 videos underwent a second review by the research team to ensure the sample approach was reliable and that each video contained substantive data relevant to the study aims. After the second review, 30 vlogs met the eligibility criteria and were included in the study.

**Table 1.** Inclusion and Exclusion Criteria of YouTube Video Blogs Related to Transgender, Diet, and Nutrition

Inclusion criteria	Exclusion criteria
Vlog of person that publicly identifies as transgender	Vlog of person that does not publicly identify as transgender
Recorded in English	Recorded in a language other than English
>500 subscribers or >1000 views	<500 followers or <1000 views
Relevant and substantial nutrition/diet content	Negligible nutrition/diet content
Public videos and playlists	Private videos and playlists
Personal YouTube accounts	Organizations, corporations, etc. accounts

Vlog, video blogs.

The Institutional Review Board of the researcher's home institution deemed this study exempt from review given the public nature of the data. However, the researchers felt that the vloggers may not have expected their posts to be utilized for research purposes. Therefore, coded initials were used to anonymize the direct quotes.

### Data analysis

The constant comparative method was utilized to explore the diet and nutrition messages shared by transgender vloggers.^26^ As a method of triangulation, both members of the research team analyzed the vlogs separately to develop themes and subthemes. The researchers then convened to discuss their results until a mutual interpretation of the data was formed.^27^ Pseudoquantitative methods were used to capture the prevalence of each subtheme as a percentage over the total vlogs (*n*=30). Relevant quotes were captured verbatim to justify each subtheme.

### The research team

The research team comprised one researcher who primarily utilized qualitative and mixed methods research strategies, and one researcher who was new to qualitative research. Both researchers identify as cisgender and heterosexual; for this reason, the researchers closely followed the guidance and ethical considerations for publishing transgender research by Adams et al., and consulted with a member of the transgender community when developing the research strategy.^23^

## Results

Of the 30 vlogs, the range of views was 114–86,477 with an average of 11,758 views. The range of vlog duration was 4:13–24:51 min with an average of 10:24 min. About 63.3% of vlogs were self-published by individuals who identified as female-to-male (FtM) (*n*=19) and 36.6% identified as male-to-female (MtF) (*n*=11). Vlogs were published in YouTube between 2013 and 2018, with the majority published since 2017 (*n*=9).

**FIG. 2.**
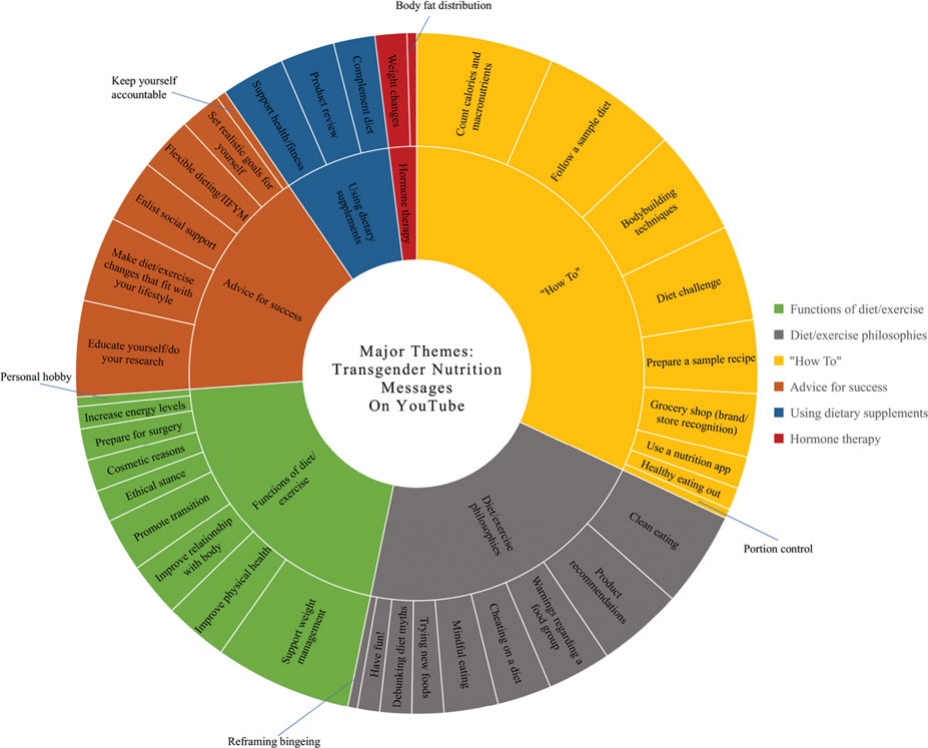
Prevalence of nutrition-related themes and subthemes shared by transgender individuals on YouTube. A pseudoquantitative analysis was conducted to code and quantify themes and subthemes.

Six major themes were generated from the constant comparative analysis (Fig. 2). These included the following: functions of diet and exercise, diet and exercise philosophies, “how to,” advice for success, using dietary supplements, and effects of hormone therapy. Each major theme was supported by 2–10 subthemes. Representative quotes from vloggers were documented verbatim.

### Functions of diet and exercise

Vloggers discussed various functions of diet and exercise in their personal lives; see Table 2 for the nine supporting subthemes. Functions of diet and exercise were specific to transgender health or applicable to the general population. The most commonly discussed function of diet and exercise was to support weight management (43%). Emma commented, “There's no tricks involved in weight loss. It's about calories…calories in, calories out.” Others shared their personal experiences with weight loss and weight gain by discussing various strategies such as calorie counting, following a vegan diet, or engaging in aerobic activity.

**Table 2.** Six Major Themes and Subthemes of the Nutrition Messages Shared by Transgender Vloggers on YouTube

Theme	Subtheme	Frequency, *n* (%)	Quotes
Functions of diet/exercise	Support weight management	13 (43)	“The fundamental principle to weight loss is calories in, calories out.” MtF LP
	Promote transition	5 (17)	“I started working out to start my transition a little bit earlier because I knew that I feel a little more comfortable. My physical body, my physical gender started to match more clearly to what I felt was aligned with my masculine gender, my male identity.” FtM AD
	Improve physical health	6 (20)	“With the vegan lifestyle I've also noticed I've been able to control my digestive system a lot better.” MtF CL
	Increase energy levels	2 (7)	“With a vegan lifestyle I have been not only able to maintain my energy levels but also increase them. I honestly have felt so energized.” MtF CL
	Prepare for surgery	3 (10)	“Today is the unofficial day that I'm starting the anti-inflammatory diet for my surgery.” FtM AD
	Cosmetic reasons	3 (10)	“My hair has gotten a lot healthier. Also, my nails have gotten a lot stronger.” MtF CL
	Improve relationship with body	5 (17)	“I know for a fact it [exercise] made me have a better relationship with my body. For sure. Now, do I have a perfect relationship with my body? No. Are there still things I wish that I could change? Yes. But, am I the most comfortable I've ever been in my entire existence with my body now? Yeah.” FtM AD
	Personal hobby	1 (3)	“I train for fun. I train because I love to do it. I train because it's my hobby.” FtM AD
	Express an ethical stance	3 (10)	“What I do is I'm ethically vegan so I eat whole, plant-based foods.” MtF BM
Diet/exercise philosophies	Clean eating	9 (30)	“I think that if you never first clean out your body or go through that process of just eating whole food, that you're never really going to learn what that's like. You're never going to learn what that feels like to have your body firing on well-prepared whole foods.” FtM RE
	Product recommendations	8 (27)	“I found my friend here: almond milk. I love the taste. It actually has 50% more calcium than regular cow milk.” MtF CL
	Warnings regarding a food group	6 (20)	“Also, like, meat and dairy cause cancer and they contribute to a lot of other health issues.” FtM TR
	Mindful eating	5 (17)	“Eat until you're satiated and you're full.” MtF BM
	Cheating on a diet	5 (17)	“Yesterday was kind of like a cheat meal which turned into a cheat night but I didn't go over my maintenance calories.” MtF LP
	Reframing bingeing	1 (3)	“I ended up bingeing in the kitchen late last night but I still tracked everything…this is to be expected when you're cutting such a high deficit, like, you're probably going to binge eventually. It's just a psychological fact unless you're extremely dedicated, binges are inevitable so you should plan for them and learn to control your binge and just psychologically rethink the way you think about a binge.” MtF LP
	Debunking diet myths	3 (10)	“You don't need as much protein as they tell you that you need. You do not. And you will not find a lot of resources out there that talk about that.” FtM RE
	Trying new foods	3 (10)	“This will be fun. It's got avocado on there. Alright, here we go…it's good!” FtM AD
	Have fun!	2 (7)	“I train for fun. I train because I love to do it. I train because it's my hobby.” FtM AD
“How to”	Follow a sample diet	13 (43)	“So this is day one. For my breakfast I'm having peanut butter on brown toast and half a grapefruit…this is what I'm having for dinner and there's 1 cup of mashed up peas, um, 3 oz of chicken, half a banana, a small apple, and a glass of water.” MtF MG
	Diet challenge	9 (30)	“I did this diet called the military diet and you're supposed to lose up to 10 lbs in 3 days.” MtF MG
	Count calories and macronutrients	13 (43)	“Started off the day with a little macro friendly breakfast sandwich, which was a 100% whole wheat English muffin, 3 Tbsp of egg whites, and 4 slices of some turkey lunch meat. And then a cup of black coffee. It puts my macros at 17 grams of protein, 31 grams of carbs, and 5 grams of fat. Not too bad.” FtM TR
	Bodybuilding techniques	10 (33)	“I have been sticking pretty good with my bulk, tracking everything in MyFitnessPal and really watching my macros and trying to eat as clean as I can without torturing myself.” FtM TR
	Use a nutrition-related app	3 (10)	“If you're looking for a really good macro calculator, muscleforlife.com macro calculator has a great one on there.” FtM RE
	Grocery shop	6 (20)	“Grocery shopping was really fun, but sadly the vegan alternative section in my local grocery store are kind of lacking. Headed to the checkout. Loaded up on mostly vegetables, a little bit of the convenience frozen stuff. I also found some vegan protein powder.” FtM TR
	Prepare a sample recipe	7 (23)	“I'll put them [sweet potato fries] in the oven at 400 for like 11 to 16 min and then flip them.” FtM AD
	Choose healthy restaurant options	2 (7)	“Yo just got out of Chipotle, had my very first every sofritas burrito which is a shredded spiced tofu mix to replace the meat.” FtM TR
	Portion control	1 (3)	“So I mix these two creamers together right here. And I used a Tbsp to actually measure.” FtM AD
Advice for success	Educate yourself/do your research	9 (30)	“Make sure that you go and do your own research. Talk to your doctor. Talk to your dermatologist or endocrinologist and get more information.” MtF CL
	Make diet/exercise changes that fit with your lifestyle	8 (27)	“Figure out what works with you and stick with that until you can add more on top of it.” FtM TR
	Set realistic goals for yourself	4 (13)	“Set a specific goal. For example, say you want to go to the gym three times a week? That's your goal. So do that for a couple weeks until that's a habit for you and it's not a struggle to get into the gym, and then, once you get that down, implement something else.” FtM TR
	Flexible dieting/IIFYM	5 (17)	“Some days I'm a lot more strict, and then some days, I'm like, you know.”
			“I'm sticking to an ‘If It Fits Within Your Macros Diet’ and recording everything in MyFitnessPal.” FtM TR
	Enlist social support	6 (20)	“The big reason we joined the vegan movement is because of my mom's health issues, with having a lot of internal things we've decided and after looking into it, going vegan would be a good option. So she's on board too, we're doing it together…teamwork makes the dream work.” FtM TR
	Keep yourself accountable	1 (3)	“My next tip is that you should find a way to keep yourself accountable. If I hadn't blogged through my first week, I probably would have broke.” FtM TR
Using dietary supplements	Support health/fitness	6 (20)	“I'm transgender. I take testosterone, so I have to really be aware of my heart and not do anything that's too hard on my heart, and I know that fish oils have omegas.” FtM TR
	Complement diet	4 (13)	“These are usually things that, not that they can't be found in food, but usually it's like, to get the amount of that thing that you would need to help, um, us humans, like these days, we're just not eating the foods that get this type of stuff.” FtM AD
	Product review	5 (17)	“I just look at how many grams of protein it has, how much sugar it has, and how it tastes.” FtM TR
Effects of hormone therapy	Weight changes	3 (10)	“Don't freak out. It's ok. Your body is going to gain some weight.” FtM AD
	Body fat redistribution	3 (10)	“The first thing that happens with, as far as weight gain or loss or whatnot is that your body is going to start redistributing fat. And that's because estrogen, which is the main hormone in female-bodied people, it carries fat in different places, right, more of the lower bum, it carries it on your thighs, and a lot around the hips and lower back area..testosterone carries fat mostly in the lower belly.” FtM AD

FtM, female-to-male; IIFYM, if it fits within your macros; MtF, male-to-female.

Relatedly, diet and exercise were discussed as a function to promote transitioning in 17% of the vlogs. Natural transitioning was described as the process of changing one's body toward a more masculine or feminine gender expression without the use of hormones or surgery. Vloggers explained how exercise in particular was helpful in supporting a natural transition. AD explained, “I started working out to start my transition a little bit earlier because I knew that I would feel a little more comfortable. My physical body, my physical gender started to match more clearly to what I felt was aligned with my masculine gender, my male identity.” Diet and exercise as a function to promote passing or a natural transition was distinct from general comments on weight management in that vloggers associated certain modifications with a more feminine or masculine gender expression.

The third subtheme specific to transgender health was diet and exercise as a means to prepare for gender-affirming surgery (10%). AD shared multiple videos tracking his experience with an “anti-inflammatory diet for surgery,” which included a diet based on fruits, vegetables, beans, and lean protein. He explained, “Overall I just feel like this has helped me prep for surgery. Like, I feel good about surgery. I feel like I prepped my body as best as I can.”

Functions of diet and exercise that apply to the general population were increased energy levels (7%), improved relationship with one's body (17%), cosmetic reasons (10%), expression of an ethical stance (10%), and personal hobby (3%).

### “How to” videos

The “how to” theme emerged from the instructional nature of numerous vlogs. The most commonly discussed topics related to following a specific diet plan. Vloggers described how to follow a sample diet (43%), count calories and macronutrients (43%), and use a nutrition-related app to track progress (10%). Techniques for communicating these messages included a visual representation of the food eaten, stepwise instructions on inputting data into an app, and narration of the nutrient content. For example, TR shared a “full day of eating video” that captured all food and beverages consumed over the course of 24 h and the corresponding nutrient content through the MyFitnessPal app.

Calorie and macronutrient counting was used to communicate body building techniques among both FtM and MtF bloggers (33%). The term “bulking” was used to describe weight gain through strength training and increased calorie consumption; the term “cutting” was used to describe weight loss through aerobic activity and decreased calorie consumption. This method was also utilized to illustrate a particular diet challenge (30%), such as a raw vegan, ketogenic, anti-inflammatory, or Paleo diet. Vloggers identified various reasons for starting a diet challenge, such as weight loss or gain. CL noted, “Sometimes we have to challenge ourselves.”

“How to” videos also included practical skills such as recipe preparation (23%), grocery shopping (20%), choosing healthy restaurant options (7%), and portion control (3%). Vloggers often integrated “how to” messages into descriptions of a sample diet. For example, TR recorded his grocery shopping and restaurant meal choice while following a vegan diet.

### Diet and exercise philosophies

The theme of diet and exercise philosophies was identified as vloggers shared their personal approaches to food and nutrition, including both what to eat and how to eat. Subthemes were “clean eating,” which was used to characterize a diet high in whole, minimally processed foods (30%), product recommendations based on nutritional value and personal preferences (27%), and warnings regarding a food group based on personal reactions to foods such as dairy, meat, and alcohol (20%).

Vloggers also addressed approaches to eating such as mindful eating, which was characterized as eating in response to internal hunger and satiety cues (17%). Others discussed cheating on a diet by normalizing “cheating” with examples of deviations from their diet challenges (17%). Relatedly, one vlogger reframed binge eating by describing her proclivity to eat more in the evening as a natural response to consuming insufficient energy during the daytime (3%).

Vloggers spent time debunking common diet myths that have emerged among the online transgender community such as those related to soy intake and protein needs (10%). This often coincided with the subtheme of educating yourself, where vloggers asserted their positions but also encouraged viewers to research the topic themselves.

Finally, vloggers advocated for trying new foods (10%) and having fun (7%). AD captured his first taste of a new dish featuring avocado, and later commented on his approach to exercise as: “I train for fun. I train because I love to do it. I train because it's my hobby.”

### Advice for success

Next, vloggers gave several pieces of advice for success in meeting personal diet and exercise goals. The most commonly discussed advice that emerged from 30% of the vlogs was to do research and educate oneself. Some recommended doing research in general, whereas others recommended specific resources. For example, when discussing how to transition to a vegan diet, CL advised, “Start doing some research. Start learning about different products that you can substitute.” TR provided more specific advice geared toward transgender men by providing specific book recommendations.

The second piece of advice observed in 27% of the vlogs was to make diet and exercise changes that fit within one's lifestyle. This was described as “You have to do what works for you” by CL and “If you want to get fit and you want to stick with it, you're going to have to do it your way” by TR. RE provided more specific advice on macronutrient distribution. Relatedly, the advice to set realistic goals was observed in 13% of the vlogs. This was described by CL as making changes “little by little” and by TR as maintaining a change until it is “not a struggle” and then implementing an additional goal.

The concept of flexible dieting or “if it fits within your macros (IIFYM)” was cited in 17% of the vlogs. This captures the trend of allowing liberties within one's food choices while still meeting target energy and macronutrient distribution goals. TR explained his use of MyFitnessPal to track his calorie and macronutrient distribution and named “IIFYM” as his dietary approach. He also provided a counterpoint to the diet by explaining, “You can make unhealthy food fit into your macros but…you're going to feel bad.”

Finally, the advice to enlist social support and keep yourself accountable emerged from 20% and 3% of the vlogs, respectively. Vloggers discussed the positive impact of their partners or family members in meeting their health and exercise goals. For example, AD commented on his wife's support in following an anti-inflammatory diet before his surgery. TR discussed how both he and his mother decided to try a vegan diet together. He also noted the positive impact of blogging on keeping himself accountable to a new diet: “If I hadn't blogged through my first week, I probably would have broke.”

### Using dietary supplements

Dietary supplement use was discussed in the context of supporting overall health or fitness goals (20%) and complementing one's diet (13%). Vloggers connected specific health concerns to their personal supplement choices. For example, TR explained his decision to take a fish oil supplement given the impact of testosterone therapy on cardiovascular health.

Vloggers also published reviews of dietary supplements based on their personal experiences (17%). In these videos, vloggers reviewed sensory qualities such as taste, texture, and smell, and the nutritional value of the product. Vloggers advocated for certain products and disparaged others.

### Effects of hormone therapy

Finally, vloggers addressed effects of hormone therapy related to weight changes (10%) and body fat redistribution (10%). Vloggers related the impact of estrogen or testosterone therapy to physiology changes associated with gender-specific body types. For example, AD discussed how estrogen may promote adiposity in different body parts such as the hips and buttocks. Vloggers also took time to reassure viewers that the effects of hormone therapy related to body weight and composition were normal and to be expected. AD encouraged, “Relax, don't worry. Keep going to the gym; keep eating right; keep taking your hormone dose…”

## Discussion

Nutrition care guidelines for the transgender population do not exist, but transgender individuals are seeking diet and nutrition information. The findings of this study suggest that social media is a key source for communicating diet and nutrition information among the transgender community. Consistent with Capurro et al.,^20^ social media platforms may support the expression of stigmatized or vulnerable voices as evidenced by the candid sharing of personal experiences, opinions, and values.

Fox and Ralston described SNS as informal learning environments for the lesbian, gay, bisexual, transgender, and queer community, especially as teaching platforms where members can share their own experiences with others.^17^ We found this to be consistent with the findings of this study in that all videos shared a teaching element either by direct instruction or modeling. The themes of “how to” and “advice for success” directly supported the educational nature of the vlogs.

Relatedly, multiple vloggers emerged as social media micro-celebrities as evidenced by single video viewership upwards of 80,000 or channel subscribership upwards of 250,000. These vloggers are viewed as gurus among the transgender community and speak powerfully from their firsthand experiences. We are compelled to note that although some of the nutrition messages shared were scientifically sound, not all messages were evidence based. The subtheme of “warnings regarding a food group” (20%) captured multiple instances where inaccurate information was shared. For example, the broad claim that dairy causes cancer is not supported by scientific research. As suggested by Blotner and Rajunov,^15^ these instances may provide an opportunity for health professionals to engage with the online transgender community, build trust, and debunk health myths.

The generated themes are suggestive of the nutrition-related topics that are important to the transgender community. Some topics apply to both transgender and cisgender individuals, such as weight management or practicing portion control. Other topics are specific to transgender health, such as diet and exercise to promote transition or the effects of hormone therapy on body weight and composition. Future research may directly investigate the nutrition-related topics that are of greatest importance to the transgender community.

Within the sample, the number of vlogs published by FtM (63.3%) versus MtF (36.6%) individuals was notable. Of the top 10 videos in terms of viewership, 9 were published by FtM individuals and only 1 was published by an MtF individual. Popularity discrepancies may be explained by the gendered nature of different body types and the utilization of diet and exercise to achieve these. Another consideration is the perceived safety of presenting on a public forum as a transgender male versus a transgender female.

### Limitations

Limitations to this study centered on the use of social media as a data source. This method did not represent those with limited ability to produce videos, those without Internet access, or older populations who may not relate to social media as a communication platform. Furthermore, this sample did not include vloggers who identify as nonbinary or genderqueer. Community representatives were not consulted throughout all stages of the research project. Future projects should consult with community representatives as much as possible, including the design, interpretation of results, and construction of the article.

### Future research

Further research is needed to fully understand how and where transgender, nonbinary, and genderqueer individuals seek nutrition information, such as other social media platforms, websites, health care providers, or community resources. Although the findings of this study suggest the nutrition-related topics that are of most importance to the transgender community, additional studies are needed to explore and quantify these areas of interest. Future projects might compare nutrition-related messages of transgender vlog content to nutrition information on YouTube as a whole, including cisgender bloggers. Attention to these topics will be critical in future studies that honor the interests and concerns of the transgender population.^23^

## Conclusions

Nutrition-related messages are shared among the online transgender community through Youtube. The major themes generated from this netnography reflect topics of interest and expressed needs of transgender individuals. Health care providers may actively engage with online communities. The power of peer-to-peer education further demonstrates the opportunity for health care providers to collaborate with content creators to improve health literacy, build trust, address questions, and provide a source of accurate and evidence-based information.^15–20^ Further research on the diet and nutrition considerations for transgender individuals should reflect the interests and expressed needs of the transgender community.

## Abbreviations Used

FtM: female-to-male
IIFYM: if it fits within your macros
MtF: male-to-female
SNS: social networking site
vlogs: video blogs
